# Amaranthus Biochar-Based Microbial Cell Composites for Alleviation of Drought and Cadmium Stress: A Novel Bioremediation Approach

**DOI:** 10.3390/plants12101973

**Published:** 2023-05-13

**Authors:** Tripti, Adarsh Kumar, Maria Maleva, Galina Borisova, Mani Rajkumar

**Affiliations:** 1 Laboratory of Biotechnology, Institute of Natural Sciences and Mathematics, Ural Federal University, 620002 Ekaterinburg, Russia; maria.maleva@urfu.ru; 2 Department of Experimental Biology and Biotechnology, Ural Federal University, 620002 Ekaterinburg, Russia; G.G.Borisova@urfu.ru; 3 Department of Environmental Sciences, Bharathiar University, Coimbatore 641046, India; mraaj13@yahoo.com

**Keywords:** *Serratia* sp., *Pseudomonas* sp., plant-growth-promoting rhizobacteria, water deficiency, heavy metal, biochar-based biofertilizer, *Brassica napus* L., morphophysiological parameters

## Abstract

Metal contamination coupled with aridity is a major challenge for remediation of abiotic stressed soils throughout the world. Both biochar and beneficial bacteria showed a significant effect in bioremediation; however, their conjugate study needs more exploration. Two rhizobacteria strains *Serratia* sp. FV34b and *Pseudomonas* sp. ASe42b isolated from multi-metal and drought stressed sites showed multiple plant-growth-promoting attributes (phosphate solubilization, indole-3-acetic acid, siderophore, and ammonia production). Both strains were able to tolerate a high concentration of Cd along with being resistant to drought (−0.05 to −0.73 MPa). The seldom studied biomass of *Amaranthus caudatus* L. was used for biochar preparation by pyrolyzing it at 470 °C for 160 min under limited oxygen and then using it for the preparation of biochar-based microbial cell composites (BMC)s. To check the efficiency of BMC under Cd stress (21 mg kg^−1^ soil) and drought, a pot-scale study was conducted using *Brassica napus* L. for 47 days. Both the BMC5 (Biochar + *Serratia* sp. FV43b) and BMC9 (Biochar + *Pseudomonas* sp. ASe42b) improved the seed germination, plant biometrical (shoot and root biomass, length of organs) and physiological (photosynthetic pigments, proline, malondialdehyde, and relative water content) parameters under drought (exerted until it reaches up to 50% of field capacity) and Cd-spiked soil. However, for most of them, no or few significant differences were observed for BMC9 before and after drought. Moreover, BMC9 maximized the Cd accumulation in root and meager transfer to shoot, making it a best bioformulation for sustainable bioremediation of Cd and drought stressed soils using rapeseed plant.

## 1. Introduction

Cadmium is a highly mobile toxic heavy metal that causes harmful effects in humans and detrimental effects in plants. Being a naturally occurring element, it is released into the environment through human activities such as mining, industrial processes and the use of fertilizers and pesticides [1,2,3]. The problem of cadmium contamination in agricultural soil is particularly acute in areas where farming is intensive and there is a high demand for food production. It can quickly accumulate in soil and water and can contaminate food crops over time, posing a significant threat to consumer health. In addition, limited water for irrigation is another major challenge for arid and semi-arid regions, which causes stunted plant growth and loss in productivity. Many abiotic stress factors such as moisture content, temperature, and water availability are regulated by drought. Moreover, when drought integrates with metals, it exerts severe plant developmental issues. When both factors are prominent, they adversely affect plant physiological and biochemical processes which could lead to growth, biomass, and yield loss [4,5]. Many drought-prone regions such as different parts of China, Africa, Europe, and the Mediterranean shrubland, reported dynamic changes in the metal concentration in soil and its subsequent transfer in plants due to drought stress [2,6,7,8]. They severely affect seed germination, leaf water content, photosynthetic activity, chlorophyll content, and organelle structure and accelerates senescence [9]. Both abiotic factors, drought and Cd cause the accumulation of reactive oxygen species and induce oxidative stress in plant cells [9,10,11]. Therefore, it is a significant concern in many parts of the world, particularly in developing countries where soil and food contamination monitoring is limited. This highlighted the need to effectively manage drought and cadmium levels in the soil to protect human health and the environment.

Plant-growth-promoting (PGP) rhizobacteria are beneficial microorganisms that colonize plant roots and promote plant growth through various mechanisms [12,13,14,15]. The PGP rhizobacteria, which manage to colonize in abiotic and biotic stressed soil, become resistant and tolerant to such factors and thus help in plant growth and development. PGP rhizobacteria often show multiple attributes to overcome such stresses, including nutrient cycling, biological control of plant pathogens and induction of systematic resistance. Many PGP rhizobacteria produce plant growth hormones such as indole-3-acetic acid (IAA), cytokinins and gibberellins, which can stimulate plant growth and development [12]. Some PGP rhizobacteria are capable of solubilizing and mobilizing nutrients from the soil such as phosphorus, nitrogen and iron, which can be taken up by the plants and thus enhancing their growth and biomass production [13,14,15]. Furthermore, numerous PGP rhizobacteria have demonstrated biological control activity against plant pathogens, such as fungi and bacteria, by producing antibiotics, siderophores, and lactic enzymes [15,16]. Thus, they play a vital role in plant tolerance by inducing systematic resistance against biotic (pathogenic bacterial and fungal attack) and abiotic stress (drought, salinity, heavy metals). Apart from PGP characteristics, these rhizobacteria are well-identified for plenty of potential for bioremediation of metals from soil (through biosorption of metal ions by their functional anionic groups) and defense responses to drought [2,17,18]. 

Despite the bioremediation potential of these PGP rhizobacteria, their survival decreases with time and thus requires a suitable carrier. Biochar (BC) is a carbonaceous material that has micro-porous structure and can provide aeration and retain moisture for higher growth of microorganisms [19,20]. BC possesses both micro- and macronutrients which further help in the nourishment of plants. More importantly, it possesses multiple functional groups which help in the biosorption of multiple metals and sequestering them into their complex matrix for a longer duration, thus reducing its bioavailability [21]. Altogether, because of the presence of micro-porous structure and metal absorbing capacity, its association with beneficial PGP rhizobacteria will lead to the development of effective BC-based microbial cell composite (BMC) which can help both in metal and drought alleviation. Most of the researches are mainly reported for salt and/or drought alleviation using PGP rhizobacteria, however seldom are results discussed for alleviation of both metal and drought using PGP rhizobacteria. Moreover, the use of BC as solid carrier material for bioremediation under such abiotic stress conditions is still limited. *Amaranthus caudatus* L. is a fast growing, high biomass lignocellulosic plant widely cultivated for seed and edible leaves [22,23], however their stem remains a waste and becomes a potential source for biochar and bioenergy production. To date, research has yet to be carried out to utilize the stem of *A. caudatus* to produce biochar. 

The aim of the present research was to: (a) isolate and identify the drought- and Cd-tolerant rhizobacteria, (b) assess the PGP attributes of rhizobacteria, (c) assess the effect of Amaranthus BC-based BMC on pigment content, proline, lipid peroxidation, and growth of *Brassica napus* L. (rapeseed) grown under Cd influenced soil before and after drought, and (d) assess Cd and drought alleviation potential of BMC using *B. napus*.

## 2. Results

### 2.1. Isolation and Identification of Rhizobacteria

Both studied plants, *Festuca valesiaca* Schleich. ex Gaudin (FV) and *Artemisia sericea* Weber ex Stechm. (AS), harbor significant population of rhizobacteria, i.e., 18.3 × 10^5^ and 0.5 × 10^5^ cfu g^−1^ of dry soil on LB agar plates which reduced significantly to 0.3 × 10^2^ and 0.8 × 10^2^ cfu g^−1^ of dry soil after supplementing with 10 mg Cd L^−1^, respectively. Furthermore, out of ten Cd-tolerating fast-growing rhizobacteria from each rhizosphere soil, a total of three and five prominent isolates from FV and AS rhizosphere, respectively, showed drought resistance to 5% (−0.05 MPa) of polyethylene glycol (PEG6000, Merck, Banglore, India) concentration. Finally, two rhizobacteria strains, FV34b and ASe42b, which showed maximum growth and Cd tolerance along with Ni and Cu and drought resistance, were selected from FV and AS plants, respectively, and further studied for identification using morphological, physiological and biochemical tests (Table 1). The strain FV34b was wavy, beige, bulging, translucent, and irregular, whereas strain ASe42b was smooth, milky, flat, translucent and round and both strains belong to the Gram-negative group. 

The 16S rRNA gene sequencing of both strains showed maximum similarity with *Serratia plymuthica* (97.10%, 1600 bp) and *Pseudomonas koreensis* (97.16%, 1597 bp) reported in the NCBI database. Therefore, the strains were finally identified as *Serratia* sp. strain FV34b and *Pseudomonas* sp. strain ASe42b and were deposited in NCBI Genbank under accession number OQ866540 and OQ866539, respectively.

### 2.2. Minimum Inhibitory Concentration, Drought Resistance, and PGP Attributes under Cd and Drought Stress

To understand the maximum metal tolerance potential, both strains were tested with increasing concentrations of Cd, Ni, and Cu. As a result, both strains tolerated a maximum of 250 mg Cu kg^−1^ and 20 mg Cd kg^−1^, whereas it was 250 and 10 mg Ni kg^−1^ for strain FV34b and ASe42b, respectively (Table 2). Multiple metal tolerance of both strains reflects their higher ability to survive under unfavorable stress conditions.

Drought resistance by both strains was performed under low (−0.05 Mpa), and medium (−0.15 to −0.49 MPa) to moderately high (−0.73 MPa) osmotic potential. Strain ASe42b showed higher resistance compared to strain FV34b and no stress was observed even at a maximum tested concentration of 30% of PEG6000. However, a slight decrease in the growth of strain FV34b was observed at 25% of PEG5000 concentration (Table 1). 

Both strains were able to produce IAA and ammonia, and solubilize inorganic tri-calcium phosphate without facing any abiotic stress. The strain ASe42b was also able to produce siderophore. An increase in IAA and siderophore production with no significant adverse effect on other PGP attributes was observed for strain ASe42b, whereas a slight decrease in ammonia but increased siderophore production were observed for strain FV34b under Cd + Drought stressed conditions. Surprisingly, no hydrogen cyanide production, an essential attribute for fighting against soil pathogens, was observed for either strain under normal and abiotic stressed condition (Table 2).

### 2.3. Biochar Preparation, BMC Development and Seed Germination

The Amaranthus biochar was prepared in a self-developed pyrolysis retort using an indirect heating method. The burning of feedstock produced huge heat energy inside the retort, caused slow pyrolysis and resulted in the generation of quality carbonaceous material called “Biochar”. The pH of the BC was found to be alkaline (10.10 ± 0.25) with high EC (10.01 ± 0.20 mS cm^−1^), and the water holding capacity (WHC) was 56.50 ± 4.55%. The bioavailable concentration of Cd and Ni in the BC was found to be below the detection limit, whereas the Cu content was 2.05 ± 0.20 mg kg^−1^. Elemental analysis depicted the percentage of CHN/O, which ranged between 49.50 and 52.25 for C, 2.50 and 2.99 for H, 0.95 and 1.12 for N and 40.77and 44.25 for O. A high O/C ratio of 0.83 showed higher stability and heavy metal adsorption potential of prepared biochar, whereas a low H/C of 0.05 showed a high level of carbonization of biomass. In spite of the many beneficial properties of biochar, these may cause detrimental effects on PGPRs because of the presence of toxic metals and pathogens, which was found to be limited in the present research. 

Seed germination percentage was maximum for both 2.5%BMC treatments and found to be in the order of BMC9 (66.67 ± 3.33) = BMC5 (66.67 ± 2.22) > (56.56 ± 2.15) > BC (50.77 ± 2.22) > Cd (43.44 ± 3.45) which showed positive effectiveness of prepared BMCs for germination of *B. napus* seeds. In addition, the maximium rate of seed germination was demonstrated by positive control which was non-significantly different from Cd + BMC9 (3.99 ± 0.25). The minimum rate was under negative control with Cd treatment (3.15 ± 0.11). An inappreciable decrease in the rate of seed germination demonstrated the exiguous toxic effect of Cd on the seeds during the germination period.

### 2.4. Plant Growth Experiment

To study the effect of Cd and drought on biomass, metal accumulation, and morphophysiological parameters, *B. napus* was grown for 47 days at a pot-scale level under five different treatments: Cd (negative control), Cd + 2.5%BC, Cd + 2.5%BMC5 and Cd + 2.5%BMC9 along with positive control (C) without Cd in a controlled condition and evaluated for above-said parameters before and after drought.

#### 2.4.1. Biometric Growth Parameters and Cd Accumulation in *B. napus* before and after Drought

**Figure 1:**
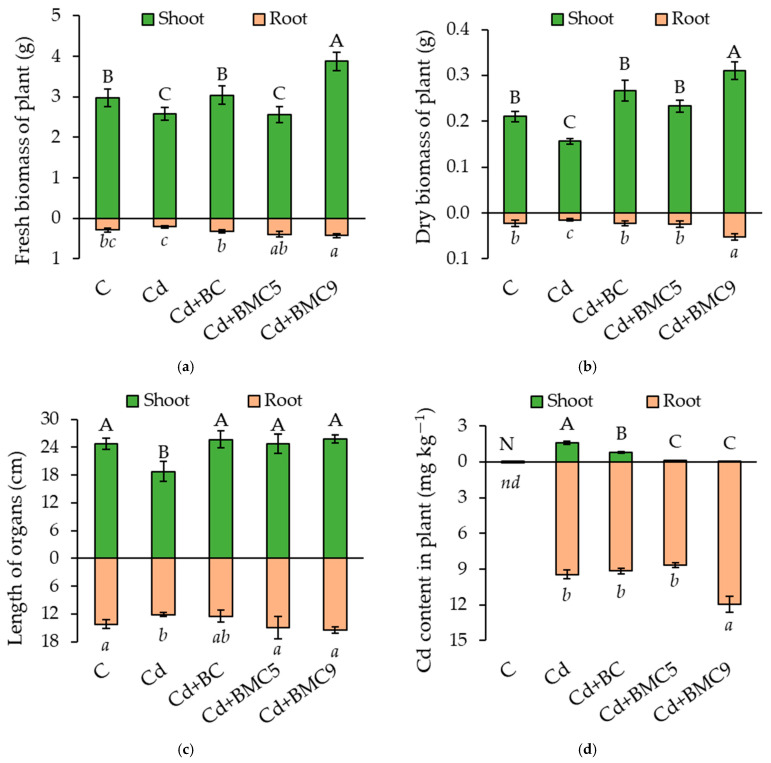
Biometrical parameters of rapeseed: (**a**) Dry biomass, (**b**) Fresh biomass, (**c**) Shoot and root length, and (**d**) Cd content in the shoot and root of plants at harvest. Capital and small letters represent significant differences between the treatments at *p* < 0.05. Data are presented as mean ± SD (*n* = 4). C: control; BC: biochar; BMC5: biochar + *Serratia* sp. FV34b; BMC9: biochar + *Pseudomonas* sp. ASe42b. Nd/(*nd*): not detected.

Application of amendments improved the root and shoot length, fresh and dry biomass of rapeseed. Both BC and BMC significantly improved the total biomass, however, significant decrease was observed for only Cd-treated plants. Maximal results were observed for BMC9-treated plants which enhanced total fresh and dry weight (DW) by 1.8 and 2.8 times as compared to Cd + Drought treatment, respectively (Figure 1a,b). Cd alone caused a negative effect on the growth of aboveground and underground organs of rapeseed, which was reflected by a significant decrease in the length of both shoot and root by 32% and 17%, respectively (Figure 1c). Application of both BC and BMCs kept the length of the *B. napus* organs at the level of positive control. 

Cadmium content in the shoot and root of *B. napus* was determined with and without Cd contaminated conditions (Figure 1d). With an increase in rapeseed biomass, the Cd accumulation also increased and reached the maximum in the root of BMC9-treated plants with minimum accumulation in the shoot. No cadmium was detected for the positive control plant, whereas significantly high Cd content in negative control was observed. Application of BMC9 significantly enhanced Cd accumulation in root while both of BMCs meager in aerial parts showed Cd biosorption property of isolate ASe42b which was used for the preparation of BMC9.

#### 2.4.2. Physiological Parameters of *B. napus* before and after Drought

**Figure 2:**
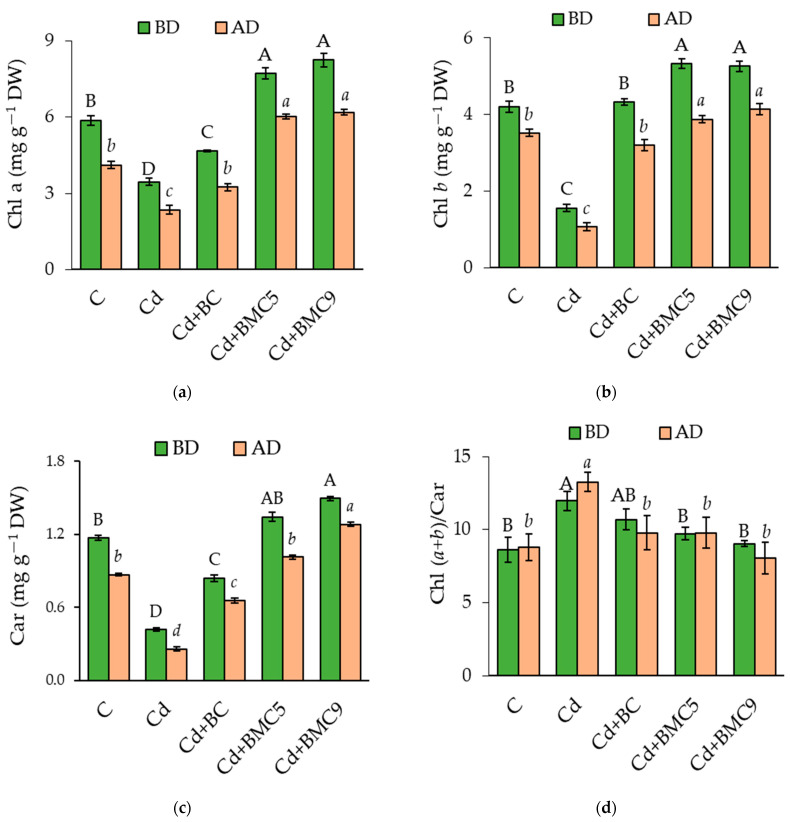
Photosynthetic pigment content: (**a**) chlorophyll *a* (Chl *a*), (**b**) chlorophyll *b* (Chl *b*), (**c**) carotenoids (Car), and (**d**) ratio Chl (*a + b*)/Car in the leaves of rapeseed before drought (BD) and after drought (AD). Capital and small letters represent significant differences (*p* < 0.05) between the treatments BD and AD, respectively. Data are presented as mean ± SD (*n* = 6). C: control; BC: biochar; BMC5: biochar + *Serratia* sp. FV34b; BMC9: biochar + *Pseudomonas* sp. ASe42b.

Presence of only Cd in soil resulted in a sharp decrease in 1.6, 2.7, and 2.8 times in chlorophyll (Chl) *a*, *b* and carotenoid (Car) content in rapeseed leaves, respectively, as compared to positive control (without Cd). In contrast, application of 2.5% BC significantly improved the pigment content (Figure 2a–c). However, a more remarkable improvement was observed when treated with BMC and best result was found to be for BMC9-treated plants. Further, drought exertion decreased all pigments content for all the treatments including positive control which was counter-balanced by application of BMC. For Chl *a* and *b*, a maximum decrease in its percentage before and after drought was observed for only Cd-treated soil (Figure 2a,b). In most cases, application of BMC increased the content of the pigments and was found to be significantly higher or similar to that of the positive control. The significant increase in the Chl (*a* + *b*)/Car ratio was observed only in the presence of Cd before and after drought (Figure 2d).

**Figure 3:**
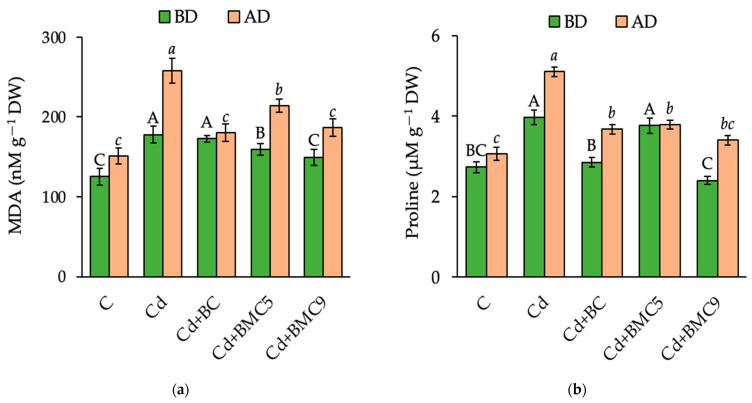
The content of: (**a**) malondialdehyde (MDA), and (**b**) proline in leaves of rapeseed before drought (BD) and after drought (AD). Capital and small letters represent significant differences between the treatments (*p* < 0.05) BD and AD, respectively. Data are presented as mean ± SD (*n* = 6). C: control; BC: biochar; BMC5: biochar + *Serratia* sp. FV34b; BMC9: biochar + *Pseudomonas* sp. ASe42b.

The malondialdehyde (MDA) content showed the maximum increase in lipid peroxidation due to the presence of Cd especially after drought. However, it reduced after the application of BC (by 43%), BMC5 (by 21%), and BMC9 (by 38%) (Figure 3a). A non-significant difference was observed between the positive control and BMC9-treated plants both before and after drought treatments, justifying the stress alleviation potential of the prepared BMC9. 

Proline content showed the application of Cd enhanced the abiotic stress in all treatments and the greatest effect was observed for only Cd-treated soil, which diminished by 28 and 39% after the application of BC and BMC9 before drought, respectively. Proline, an important drought stress indicator, increased after exertion of drought as compared to before drought and its maximum increase was observed for only Cd + Drought-treated soils. However, a 28% and 33% decrease was observed for BC and BMC9 compared to Cd + Drought, respectively. Application of BMC9 significantly reduced the proline content both before and after drought conditions (Figure 3b).

**Figure 4:**
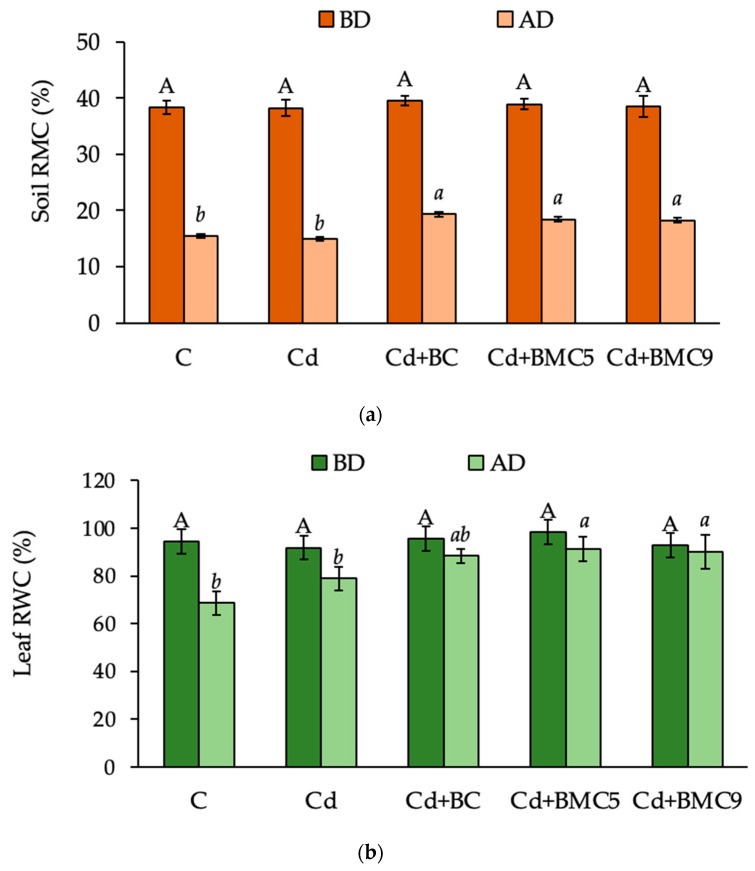
(**a**) Soil relative moisture content (RMC) and (**b**) Leaf relative water content (RWC) in *B. napus* before drought (BD) and after drought (AD). Capital and small letters represent significant differences between the treatments (*p* < 0.05) BD and AD, respectively. Data are presented as mean ± SD (*n* = 4). C—control; BC—biochar; BMC5—biochar + *Serratia* sp. FV34b; BMC9—biochar + *Pseudomonas* sp. ASe42b.

The soil relative moisture content (RMC) was maintained similarly for all treatments before exertion of drought to the plants and no significant differences were observed in their leaf relative water content (RWC) at the start of drought experiment (37th day) suggesting a minimal effect of Cd on the rapeseed plants (Figure 4a,b). Three consecutive drought shocks resulted in decrease in leaf RWC for all treatments however a significant decrease was observed for positive and negative control elucidating the adverse effect of drought stress (Figure 4b). An increase of 12%, 16%, and 14% in leaf RWC was observed for BC, Cd + BMC5 and Cd + BMC9 as compared to negative control (Cd). Application of BC and BMCs helped to retain maximum soil RMC by increasing it by 29% and 23% after drought as compared to negative control (Figure 4a) and thus positively affect leaf RWC (Figure 4b).

## 3. Discussion

The PGP rhizobacteria are widely studied due to their multiple PGP attributes under both abiotic and biotic conditions, as these establish beneficial interactions with host plants [4,13]. In the present research, two rhizobacteria genera *Serratia* sp. strain VC34b and *Pseudomonas* sp. strain ASe42b isolated from semi-arid and heavy-metal-contaminated sites showed great potential to tolerate multiple metals including Cd and moderate to high levels of drought conditions. The isolated strains showed positive results when tested for IAA synthesis, phosphate solubilization, siderophore, and ammonia production in non-stressed conditions. The effect of Cd + Drought further enhanced the IAA (helps in root elongation for higher uptake) and siderophore production (for multiple metal chelation) of the strains, possibly nullifying the adverse effect and providing sufficient nutrients for the development of the plants. A significant concentration of phosphate is present in soil; however, its availability remains limited because of the inorganic nature of phosphate. Both PGP rhizobacteria strains were able to solubilize the inorganic tri-calcium phosphate from soil and increase its availability, which further helped plants in combating nutrient deficiency. Production of siderophores was also reported by many researchers which helped in the chelation of Fe and Cu along with other essential metals and thus increased its availability for plant growth and development [15,24,25]. In the latest review, both *Pseudomonas* sp. and *Serratia* sp. were recognized for their PGP attributes and drought stress alleviation [26]. Under Cd + Drought treatment, despite a decrease in ammonia concentration by VC34b, both strains produced significant ammonia, which further supplies nitrogen for root and shoot elongation in the plant. 

Since these bacteria have low survivability under stressed conditions, they can be amalgamated with carriers such as BC, which can help alleviate stresses. In the present study, Amaranthus-based BC was produced, which showed alkaline pH, high moisture and WHC. BC provides great shelter, aeration, moisture and micronutrients, which can help in longer survivability of the isolated strains which was also reported by [10,27]. The prepared BMC (mixture of BC and PGP rhizobacteria) showed a great potential to enhance seed germination under Cd and drought stress. In contrast, a decrease in its count in only Cd-treated soil was observed. A decrease in the percentage of seed germination under Cd or other metal stress was previously reported by other researchers [28,29]. 

The study of physiological parameters evidenced that Cd and drought can significantly reduce the photosynthetic pigment content of the *B. napus* plant. A maximum decrease in Chl content in *Trifolium arvense* under drought + multimetal was also reported by Ma et al. [30]. Al Mahmud et al. [31] also reported that treatment of *Brassica juncea* seedlings with Cd led to a decrease in Chl content. Hasanuzzaman et al. [32] investigated the effect of drought stress (10% and 20% PEG) on *B. napus* and found that the content of Chl *a* was not significantly affected by moderate drought stress (10% PEG) treatment; however, plants exposed to severe drought stress (20% PEG) showed 52% lower Chl *a* content than the control plants. Similarly, a study with red maple (*Acer rubrum*) showed that the combined metals (Ni, Cu, Co, and Cr) and drought stress reduced plant growth (total leaf area, leaf dry matter and stem length) in an additive manner [33]. These authors explained that the combined stress reduced hydraulic conductance, xylem and leaf-specific conductivity by changing xylem structure and reducing conduit density in stems and conduit size in roots.

Application of Amaranthus BC enhanced biomass production, micronutrients and moisture; however, an increase for both shoot and root biomass was observed after the application of BMCs as compared to only BC under Cd + Drought stress conditions. Application of BMC not only helped to modify the physical properties of the soil due to BC but also enhanced the nutrient availability because of the presence of PGP rhizobacteria which exhibit multiple PGP attributes. Zhang et al. [34] also reported increased Chl content after the application of siderophore producing rhizobacteria under abiotic stressed conditions. However, the plant capability for accumulation of both macronutrients and essential trace metals decreased severely under drought conditions [35] and may be exacerbated by the presence of Cd in soil, causing lower biomass growth compared to only Cd-treated plants without drought, which was shown in the present study. 

The presence of Cd significantly increased the proline content in plants as compared to positive control and was highly aggravated under Cd + Drought treatment. A sharp increase in proline accumulation by 71% and 125%, compared to the control, was also recorded in *B. napus* seedlings subjected to 10% and 20% PEG, respectively [32]. BMC9 showed a great response in alleviating the stress possibly due to the microbial-mediated decrease in injury level in plants by synthesizing plant beneficial metabolites such as IAA, siderophores, solubilized P, etc., and through proline osmoregulation [4,36]. An increased accumulation of proline under water stressed conditions was possibly a measure to provide protection to the Amaranthus plants, which was also reported by [37]. Proline upsurge for internal detoxification was previously reported when inoculated with PGP rhizobacteria under drought and metal stress [4,38,39]. 

Similarly, abiotic stress causes severe membrane damage and MDA is a vital biomarker to find the reactive oxygen species (ROS)-mediated lipid peroxidation in plants. Metals/metalloids generate ROS in plant cells by disrupting the chloroplastic and mitochondrial electron transfer activities, as well as the peroxisomal oxidative metabolism [40]. Cadmium stress increased MDA, H_2_O_2_, and O_2_ levels in different crops [41]. The increased generation of ROS is also the inevitable consequence of a plant’s response to drought stress [26]. Many studies have reported drought-induced ROS overproduction and oxidative stress in numerous plant species [40]. For example, moderate and severe drought stress, subjected to 10% and 20% PEG, respectively caused a significant increase in MDA contents in rapeseed seedlings, by 65% and 123% higher compared to the control [32].

In the present research, an increase in the MDA content was observed under Cd stress, which increased after applying Cd + Drought. However, a non-significant difference in the lipid peroxidation was observed in BC- and BMC9-treated plants as compared to positive control, justifying the protection of plants from oxidative damage due to the presence of BC and PGP rhizobacteria under Cd + Drought conditions. Similar results were also reported by [4,42], where MDA content was reduced because of the bioaccumulation of metals and activity of antioxidants under metal + drought stressed conditions. Sabir et al. [43] also found decreased MDA and proline contents in *B. napus* under Cd stress through the treatment of *Enterobacter* sp. MN17 and paper and pulp waste biochar, and further demonstrated that the amendment of biochar, which immobilize heavy metal content in soil, reduced ROS production in plants by reducing the plant metal uptake and accumulation. Thus, the application of BMC9 (BC + *Pseudomonas* sp. ASe42b) protects against oxidative stress and membrane damage in plants by reducing MDA content and, therefore, the content of free proline probably did not change. 

The PGP rhizobacteria not only alleviated the oxidative damage to the plants, but also improved the leaf water content under Cd + Drought conditions. The presence of BC with very high WHC and porous structure could help retain high water content in soil and was found to be significantly correlated with high soil moisture in the BC- and BMCs-treated soils. Previously, Hafez et al. [44] also found that the combined treatment of rice husk and corn stalk (1:1) biochar with PGPB (*P. koreensis* and *Bacillus coagulans)* increased RWC in *Oryza sativa* L. under saline soil with water deficit by increasing soil moisture, consequently declining osmotic stress and avoiding plants from losing turgor. A decrease in leaf RWC and plant biomass could be attributed to a loss in hydraulic conductance and turgor pressure caused due to concomitant Cd and drought which was also reported by [4,45]. Presence of significant water in soil and improved leaf RWC, promote plant growth and enhanced biomass of both shoot and root. Application of metal- and drought-tolerant PGP rhizobacteria increased the RWC by influencing stomatal functioning and maintaining water loss which was also reported by [30]. The metabolites produced by PGP rhizobacteria such as siderophores, IAA, ammonia, and solubilized phosphates further help improve RWC in plants, which was also evidenced in several other studies [4,42,46].

Under metal and drought conditions, the significant accumulation of Cd in the root of *B. napus* justifies its high Cd accumulation potential along with its drought resistance ability. However, significant Cd transfer was also observed, which was alleviated after the application of BC and BMC. Maximum accumulation of Cd in roots of BMC9-treated plants with meager or minimal transfer in aerial part concludes the biosolubilizing potential of the *Pseudomonas* sp. strain ASe42b and its localization in root vacuoles, thus indicating BMC9 could be exploited in combination with rapeseed plant for sustainable bioremediation of Cd and drought stressed soils. In addition, it could also minimize the Cd + Drought problem of agricultural soil caused due by the use of fertilizers. Liu et al. [27] have also reported high Cd remediation efficiency under BMC-treated soil because of the presence of BC and PGP rhizobacteria, which provide significant aeration, surface nutrients and shelter for higher growth of PGP rhizobacteria. However, further research works including the investigation on the analysis of transcriptional variations in biochar and bacteria-treated plants under heavy metal and drought stress conditions are necessary to explore the exact molecular mechanisms involved in biochar- and bacteria-mediated alleviation of multiple environmental stresses (Cd + Drought) in plants.

## 4. Materials and Methods

### 4.1. Soil Sample Collection

The rhizospheric soil samples were collected from two semi-arid and heavy-metal-contaminated sites in the Chelyabinsk region and the Republic of Bashkortostan (Russian Federation). Two native plants *F. valesiaca* (Troitsk, Chelyabinsk Region: 54°3′22.08″ N 61°33′42.88″ E) and *A. sericea* (Uchaly, Republic of Bashkortostan; 54°18′26.51″ N 59°24′44.33″ E) were uprooted and the soils adhered to the root strands were collected by gently shaking them in Ziplock bags and them transferring them to the laboratory. The samples were refrigerated at 4 °C until further processing and analyses were carried out. Heavy-metal-tolerant culturable bacteria were isolated from the rhizospheric soils. The total metal concentration (Cd, Ni, and Cu) in soil samples was determined via digestion of soil (250 mg) with HNO_3_:HClO_4_:HF in a ratio of 5:1:1 (*v/v/v*) on a hot plate in a fume-hood. The samples were filtered using Whatman filter paper #42 (Whatman, plc, Kent, UK), diluted and analyzed using atomic absorption spectrometer AA240FS (Varian Australia Pty Ltd., Mulgrave, Victoria, Australia).

### 4.2. Isolation of Cadmium-Tolerant and Drought-Resistant Rhizobacteria

The collected rhizospheric soil of about 10 g was mixed with 90 mL of phosphate buffer (pH 6.5) and shaken at 180 rpm in an orbital shaker for 2 h at 28 °C. The sample was serially diluted and 100 µL of required dilution aliquot was spread on a Luria-Bertani (LB) agar plate supplemented with 10 mg Cd L^−1^ to isolate cadmium-tolerant bacterial strains. For the growth of culturable bacteria, the plates were incubated for the next 3 days at 28 °C in a bacterial incubator (Remi, Chennai, India). A total of five morphologically different-appearing bacterial colonies with high growth rate and size were selected for drought resistance analysis [25]. MIC was calculated by increasing the Cd, Cu and Ni concentration until their growth arrest [10]. 

The selected Cd-tolerant bacterial strains were grown on PEG6000-supplemented tryptone-yeast extract-glucose (TYEG) medium. A 100 µL of freshly prepared bacterial inoculum (OD adjusted to 10^8^ cfu mL^−1^) was inoculated in PEG supplemented TYEG medium and incubated at 28 ± 2 °C for 48 h at 160 rpm. The stress on bacterial isolates was measured at 600 nm using a multimode plate reader Infinite 200 PRO (Tecan, Grodig, Austria) under different water potentials of 5%, 10%, 15%, 20% and 25% PEG6000 [29].

### 4.3. Genetic Identification of Bacterial Strains

Different morphological, physiological, and biochemical tests were performed to preliminarily identify the rhizobacteria strains and cross-checked using Bergey’s manual [47]. The genomic DNA of the selected pure bacterial strains was isolated using DNeasy Plant Mini Kit (Qiagen, Hilden, Germany). To isolate the genomic DNA, DNeasy Plant Mini Kit (Qiagen) was used and further purified by NucleoSpin DNA clean-up kit. PCR instrument was used to amplify the 16S rRNA gene by PCR by optimizing through Maxima Hot Start Green PCR Master Mix and universal primers 27F (5′-GAGTTTGATCACTGGCTCAG-3′) and 1492R (5′-TACGGCTACCTTGTTACGACTT-3′). The PCR conditions were: initial denaturation at 98 °C for 30 s followed by 30 cycles of denaturation at 98 °C for 10 s, annealing at 55 °C for 30 s, extension at 72 °C for 30 s and final extension for 5 min at 72 °C. Agarose gel (1.5%) electrophoresis was run by adding 1 µL Gel Red dye (Biotium, Fremont, CA, USA) in 1× TAE buffer for 20 min to visualize the PCR product. The samples were further purified and precipitated by adding 3M sodium acetate and isopropanol. Agarose gel (1.5%) electrophoresis were maintained as described in our previous research [15]. Chromas software and BLASTn algorithm was run to compare with the NCBI sequences. The sequences were submitted in NCBI to receive an accession number.

### 4.4. Characterization of Plant-Growth-Promoting Attributes

Different PGP attributes were determined qualitatively such as IAA production, tri-calcium phosphate solubilization, siderophore, and ammonia production. IAA production was confirmed by appearance of pink color after adding Salkowski’s reagent [48], phosphate solubilization by appearance of yellow color after reacting with vanado-molybdic reagent [49], siderophore by orange halo zones around the bacterial colonies and ammonia production by change in color from yellow to reddish-brown [15] with and without Cd and drought stress. Overnight grown bacterial culture broth adjusted to 10^8^ cfu mL^−1^ was used for performing and analyzing all experiments in tri-replicates.

### 4.5. Biochar Preparation, Characterization, and BMC Development 

*Amaranthus caudatus* L. collected from the Botanical Garden of the Ural Federal University, Ekaterinburg was pyrolyzed at 470 °C for 160 min under a limited oxygen supply in a self-manufactured retort kiln and BC was obtained which was further used for developing BC-based BMC. The pH of BC was determined in 1:2.5 (*w/v*) ratio and WHC was calculated using Keen’s box [50]. Plant available Cd, Ni and Cu concentration in BC was determined by using pH 7.3 diethylenpentaaceticacid extractable solution and analyzed by AAS [50]. The elements CHN/O were determined by elemental analyzer (Elemental Analyzer system GmbH, Germany). The BC was sieved through 300 mesh, jam-packed in heat resistant polyethylene bags, and autoclaved for 25 min at 121 °C for three consecutive days. BMC was prepared by inoculating overnight grown (adjusted to ∼10^8^ cfu mL^−1^) pure culture of PGP and drought-tolerant rhizobacteria *Serratia* sp. FV34b and *Pseudomonas* sp. ASe42b with BC in a ratio of 4:1 (*v:w*) and named BMC5 and BMC9, respectively. Later, dried in laminar airflow until it reached a moisture content of 40% and stored in Gamma-rays sterilized Ziplock bags. 

### 4.6. Plant Growth Experiments

#### 4.6.1. Pot-Scale Study with *B. napus*

The pot-scale study was performed in a greenhouse chamber in the Laboratory of Biotechnology, Ural Federal University, Ekaterinburg. The peat soil (Cd content below detection limit of AAS; 0.04 mg kg^−1^) procured from local market was used to perform the experiment after sterilizing it twice at 130 °C for 1 h, later spiked with Cd and left for 14 days in open air for equilibration. The Cd was spiked by placing the pots in a plastic tray filled with 21 mg L^−1^ of 3CdSO_4_ × 8H_2_O solution and left overnight for capillary absorption. Ten *B. napus* seeds were sown in each pot filled with 150 mL of sterile soil. A total of 5 treatments: control (positive control), Cd (negative control), Cd + BC (Cd with 2.5% biochar), Cd + BMC5 (Cd with 2.5% of *Serratia* sp. strain FV34b-loaded biochar) and Cd + BMC9 (Cd with 2.5% of *Pseudomonas* sp. strain ASe42b loaded biochar) were prepared in two sets to analyze before and after drought stress. Seed germination was verified by sowing 10 sterile seeds in each pot. The seeds were coated with prepared BC or BMC mixed with distilled water in 1:1 ratio (*w:v*) and kept for 2 h except for positive and negative control which were soaked in water. Lastly, the seeds were sown in the pots and allowed to germinate for 7 days with a day and night regime of 14:10 h at room temperature in a green-house illuminated with 155 ± 20 µM m^−2^ s^−1^ provided via phytolamps (ULI-P10-18W/SPFR IP40). Each treatment had four replicates and the pots were kept in a completely randomized block design. The seed (seed germination is the number of seeds germinated to the total number of seeds sown × 100) with a radical >0.5 cm was considered a germinated seed. After the germination test, thinning was carried out on 7th day after sowing (DAS) and only four plants per pot were left for further growth experiments. The RMC of soil was recorded using a digital moisture probe meter by directly inserting it into the soil and distilled water was added if the moisture content fell below ∼70% of the field capacity. At 36th DAS, the plants of the first set were sacrificed, and the leaves and soil were collected to study the morphophysiological parameters and moisture content, respectively. The drought stress was applied to the second set thrice at 37th, 40th and 43rd DAS by irrigating the soil only up to 50% of their field capacity. After implementing drought, the plants were uprooted carefully at 47th DAS to study the after drought changes in the biometrical and physiological parameters.

#### 4.6.2. Biometric Growth Parameters and Cd accumulation in *B. napus*

Shoot and root fresh and dry biomass of rapeseed plants were collected to assess biometric growth parameters. The dry biomass was homogenized and wet digested using HNO_3_:HClO_4_ (5:2, *v:v*) to determine the Cd content in roots and shoots using AAS.

#### 4.6.3. Physiological Parameters of *B. napus*

Photosynthetic pigments (Chl *a*, *b* and carotenoids) were extracted in 80% cold aceton, measured at 470, 647, and 663 nm and finally calculated according to Lichtenthaler [51]. The content of lipid peroxidation products was measured based on the level of MDA produced in the reaction with thiobarbituric acid by the absorption at 532 and 600 nm [52]. Free proline content was determined by extracting it in boiling water, which was further stained using ninhydrin solution with glacial acetic acid in equal ratio at a wavelength of 520 nm [10]. Finally, all physiological and biochemical parameters were determined spectrophotometrically using multimode plate reader.

The RWC was evaluated before and after drought treatment via the floating disc method [53] and calculated according to Hellmuth Equation (1) [54]:

RWC (%) = (FW − DW)/(SW − DW) × 100(1)

where: FW—the initial fresh weight, SW—the fresh weight after 3 h saturation, and DW—the oven-dry weight.

### 4.7. Statistical Analyses

The results were reported in four-replicates unless specified and presented in mean and standard deviation (SD). Reagents were of analytical grade and distilled water was used for irrigation and chemical preparation. The normality and homogeneity of variances were verified using the Shapiro–Wilk and the Levene tests, respectively, and significant difference between treatments were determined using one-way analysis of variance followed by Tukey’s test. Different small and capital alphabetical letters indicate significant difference between treatments at *p* < 0.05. Positive control was only used to compare the effect of Cd on the *B. napus* plant in negative control. In contrast, negative control was used for comparing the other treatments before and after drought conditions.

## 5. Conclusions

Two rhizobacteria strains were isolated from two semi-arid and heavy-metal-contaminated areas which showed the potential to tolerate multiple metals and varying drought stressed condition. These strains were identified as *Serratia* sp. strain VC34b and *Pseudomonas* sp. strain ASe42b by morpholphysiological characteristics followed by 16S rRNA gene sequencing. The strains VC34b and ASe42b were found to exhibit multiple plant-growth-promoting attributes such as IAA, siderophore production, and phosphate solubilization under high Cd and moderate-to-high drought stress conditions. Biochar prepared through indirect pyrolysis from *Amaranthus caudatus* stem showed multiple beneficial physicochemical properties (high pH, carbon content and water holding capacity), along with negligible Cd, which was used for preparing microbial biomass composites (BMC)s for Cd remediation. Pot-scale experiments with Cd in soil showed an increase in shoot and root fresh and dry biomass, length, and photosynthetic pigment content in the leaves of *Brassica napus* with a decrease in stress by reducing lipid peroxidation in BMC5- (Biochar + *Serratia* sp. VC34b) and BMC9- (Biochar + *Pseudomonas* sp. ASe42b) treated plants before and after drought stress. Small or no significant differences were observed for the above stated parameters when drought was implemented along with Cd. Altogether, 2.5% of Amaranthus biochar-based BMC9 showed the greatest results for almost all of the studied parameters. It was able to concentrate maximum Cd in rapeseed root with negligible transfer in the shoot, elucidating *B. napus* as a potential plant to combat the Cd and drought stress condition. Future perspectives include the imaging of PGP rhizobacteria association with biochar, plant antioxidants and gene expression studies related to drought stressed parameters.

## Figures and Tables

**Table 1:** Physiological and biochemical parameters of *Serratia* sp. strain FV34b and *Pseudomonas* sp. strain ASe42b.

Parameters	*Serratia* sp. Strain FV34b	*Pseudomonas* sp. Strain ASe42b
**Physiological parameters**		
pH range tolerance	5.5–8.0	5.0–8.5
Optimal pH	7.1 ± 0.2	7.0 ± 0.2
Optimal osmotic tolerance (MPa)	−0.49	−0.73
Optimum temperature (°C)	28 ± 2	27 ± 2
Gram staining	Negative	Negative
Shape	Rod	Rod
Motility	Motile	Motile
**Biochemical test**		
Endospore staining	−	−
Catalase	+	+
Oxidase	−	+
Citrate	+	+
Indole	+	+
Methyl red		−
Voges–Proskauer	+	−
Urease	−	+
H_2_S	−	−
Glucose fermentation broth	+	+
Lactose fermentation broth	−	−

+: positive; −: negative.

**Table 2:** Minimum inhibitory concentration (MIC), drought resistance, and PGP characteristics of two isolated strains.

Parameters	Attributes	*Serratia* sp. Strain FV34b	*Pseudomonas* sp. Strain Ase42b
MIC (mg kg^−1^)	Cd	20	20
	Ni	250	10
	Cu	250	250
Drought resistance, PEG6000, % (Mpa)	5% (−0.05)	++	+++
	10% (−0.15)	++	+++
	15% (−0.30)	++	+++
	20% (−0.49)	++	+++
	25% (−0.73)	+	+++
PGP attributes	IAA production	+++	++
	Siderophore production	−	+++
	P-solubilization	+	+
	Ammonia production	++	+
	HCN production	−	−
PGP attributes under Cd + Drought	IAA production	++++	+++
	Siderophore production	+	++++
	P-solubilization	+	+
	Ammonia production	+	+
	HCN production	−	−

++++: Very high; +++: High; ++: Moderate; +: Low; −: No; Cd at 20 mg L^−1^ and drought at 25% PEG6000.

## Data Availability

All data are presented in the article.
